# Patient with X-linked phenotype of SCID, markedly skewed maternal X-inactivation, but normal common gamma chain (CD132) gene ORF sequence

**DOI:** 10.1186/1710-1492-8-S1-A24

**Published:** 2012-11-02

**Authors:** Anna-Claire Coleman, Andrew C Issekutz, Christie Riddell, Elie Haddad, Thomas B Issekutz

**Affiliations:** 1 Department of Pediatrics, Dalhousie University, Halifax NS, Canada; 2 Department of Molecular Diagnostics, IWK Health Centre, Halifax, NS, Canada; 3 Department of Pediatric Immunology and Rheumatology, CHU Sainte-Justine, Montreal, QC, Canada

## Background

A 7.5-month old male presented with increasing respiratory distress progressing to severe hypoxia, an erythematous scaling rash and a paucity of lymphoid tissue. Chest x-ray revealed bilateral pneumonia with diffuse alveolar opacities. He had an increased neutrophil count, normal hemoglobin and platelet count, and an absolute lymphocyte count of 4.3 x 10^9^/L decreasing to 1.8 x 10^9^/L. IgG was 1.72 g/l, IgA 0.59 g/l and IgM 1.08 g/l, but no antibody to tetanus, diphtheria or pneumococcus despite immunization. Nasopharyngeal aspirate demonstrated rhinovirus by PCR, and tracheal aspirate was positive for *Pneumocytis jiroveci* by immunofluoresence. Lymphocyte markers showed: 62% CD19^+^, 24% CD4^+^, 1% CD8^+^ cells and 0.5% NK cells. CD4^+^ cells were 90% CD45RO^+^, 8% CD45RA^+^ and 5% were CD25^+^. His cells had no proliferative response to anti-CD3 or IL-2 stimulation, a weak response to PHA, and no response to antigen or MLR. BMT was performed from a HLA-identical sister and the patient is well 7 months post BMT.

## Conclusion

The decreased and non-functional T cells, absence of NK cells and normal number of B cells, and lack of proliferation to IL-2 is typical of X-linked common gamma chain or JAK3 deficient SCID. DNA sequencing showed no sequence variants in the ORF of the common gamma chain, but the patient’s mother has an abnormal lymphocyte subset profile and maternal T cells are markedly skewed to use one X-chromosome (non-random), while B cells demonstrate random X-inactivation. Investigations are underway to assess whether this patient has an unusual form of X-linked SCID.

